# 

**Published:** 1896-11

**Authors:** 


### CONTENTS.

ORIGINAL COMMUNICATIONS—
THE EVOLUTION OF MODERN MEDICINE. By Joseph Eve Allen, M.D., Augusta, Ga.... 577
USEFUL MEDICATION IN TYPHOID FEVER. By Henry R. Slack, Ph.M., M.D., La-Grange, Ga.... 595
A FEW REMARKS PERTAINING TO DIAGNOSIS AND TREATMENT OF ORDINARY EAR AFFECTIONS. By F. Pierce Hoover, M.D., New York City.... 597
ABSTRACT OF CASES REPORTED AT MEETING OF TRI-STATE MEDICAL ASSOCIATION, OCTOBER 12-15, 1896. By R. R. Kime, M.D., Atlanta, Ga.... 600
ARTIFICIAL LOCAL ANEMIA IN SURGERY. By F. von Esmarch.... 601
CORRESPONDENCE—
NEW YORK LETTER.... 510
TETANUS AND HYDROPHOBIA.... 614
EDITORIAL—
DISPOSAL OF THE DEAD.... 615
SPECIALIZATION IN MEDICINE.... 617
SUBSCRIPTION BILLS AND AN EXPLANATION.... 618
CONTROVERSIES.... 619
TO THE MEMBERS OF THE STATE MEDICAL ASSOCIATION.... 619
SELECTIONS AND ABSTRACTS—
SKIN DISEASES AND SYPHILIS.... 620
MISCELLANEOUS.... 624
GENERAL MEDICINE AND THERAPEUTICS.... 630
MEDICAL ITEMS.... 641
BOOK REVIEWS.... 645
MEDICAL ITEMS—CONTINUED.... x, xii, xiii, xiv, xv
ADVERTISERS' INDEX TO ADVERTISEMENTS.... xvi
CLASSIFIED INDEX TO ADVERTISEMENTS.... xxxv

